# Online Quantification of Lactate Concentration in Microdialysate During Cerebral Activation Using ^1^H-MRS and Sensitive NMR Microcoil

**DOI:** 10.3389/fncel.2019.00089

**Published:** 2019-03-19

**Authors:** Yannick Crémillieux, Ursule Dumont, Leslie Mazuel, Roberto Salvati, Vanessa Zhendre, Silvia Rizzitelli, Jordy Blanc, Hélène Roumes, Noël Pinaud, Anne-Karine Bouzier-Sore

**Affiliations:** ^1^Université de Bordeaux, Bordeaux, France; ^2^UMR5255 Institut des Sciences Moléculaires (ISM), Talence, France; ^3^UMR5536 Centre de Résonance Magnétique des Systèmes Biologiques (CRMSB), Bordeaux, France

**Keywords:** barrel cortex, lactate, microdialysis, ^1^H-MRS, NMR microcoil

## Abstract

The dynamic *in vivo* profiling of lactate is of uppermost importance in both neuroenergetics and neuroprotection fields, considering its central suspected role as a metabolic and signaling molecule. For this purpose, we implemented proton magnetic resonance spectroscopy (^1^H-MRS) directly on brain microdialysate to monitor online the fluctuation of lactate contents during neuronal stimulation. Brain activation was obtained by right whisker stimulation of rats, which leads to the activation of the left barrel cortex area in which the microdialysis probe was implanted. The experimental protocol relies on the use of dedicated and sensitive home-made NMR microcoil able to perform lactate NMR profiling at submillimolar concentration. The MRS measurements of extracellular lactate concentration were performed inside a pre-clinical MRI scanner allowing simultaneous visualization of the correct location of the microprobe by MRI and detection of metabolites contained in the microdialysis by MRS. A 40% increase in lactate concentration was measured during whisker stimulation in the corresponding barrel cortex. This combination of microdialysis with online MRS/MRI provides a new approach to follow *in vivo* lactate fluctuations, and can be further implemented in physio-pathological conditions to get new insights on the role of lactate in brain metabolism and signaling.

## Introduction

Lactate, an ubiquitous metabolite in brain tissue, is known both as a metabolic end product appearing during anaerobic glycolysis and as a central player in cellular and inter-cellular metabolism (Nemoto et al., 1974; Ide and Secher, 2000; Simpson et al., 2007; Rogatzki et al., 2015). Given the diversity of observations and hypotheses on the role of lactate in brain metabolism, the knowledge of local variations in lactate concentration during brain stimulation is fundamental for a thorough understanding of its actual contribution to brain metabolism. The development of innovative techniques capable of accurately quantifying the concentration of lactate is one of the areas of research needed to better understand the role of lactate in the brain.

Since the early 90's, a lactate increase was detected in the human visual cortex following photic stimulation by localized *in vivo* NMR spectroscopy (Prichard et al., 1991; Sappey-Marinier et al., 1992). However, this kind of measurement can hardly, if at all, be done in rodents, in which the signal-to-noise ratio is insufficient to correctly detect and quantify the lactate peak. On ^1^H-NMR spectrum, lactate resonance is mainly observed through the doublet at 1.32 ppm. This doublet corresponds to the three protons of the methyl group, which form an A_3_X spin system. The methine group quartet at 4.1 ppm is typically not observed *in vivo* due to its proximity with the water peak, which is suppressed. Two major problems lead to the poor detection of lactate; first its low physiological content. Extracellular lactate concentration is around 2 mmol/L in the human healthy tissue (Langemann et al., 2001) and even lower in rodent, between 0.1 and 1.6 mmol/L (Shram et al., 1998; Mosienko et al., 2015). Second, its NMR methyl peak (1.32 ppm) is in the spectral region where large lipid resonances are present. The overlap of the lactate doublet with macromolecule peaks prevents its detection by ^1^H MRS. Several lactate editing sequences have been proposed (Hurd and Freeman, 1991). The general idea is to apply longer echo time (TE) such as the lipid signal is strongly reduced. However, this leads also to a decrease of the lactate signal. Therefore, this approach can mainly be used in pathological situations, in which the lactate concentration is high enough. In this context, despite the reliability of metabolite quantification attainable at ultra-high fields and the possibility of using spectral editing techniques, the precise quantification of lactate in the healthy brain is quite difficult. It becomes even more difficult to quantify the small lactate variations expected during brain stimulation, a constraint we also have to deal with in addition to the limited temporal and spatial resolution of MRS.

Brain-implanted microdialysis is a widely used sampling tool for monitoring the concentration of chemical species in extracellular fluids (Fellows et al., 1992; Zielke et al., 2009). Lactate concentrations can be measured by this technique collecting brain extracellular fluid (ECF) (Korf et al., 1993; Langemann et al., 2001; Zielke et al., 2009; Horn and Klein, 2010). When combined with MRS, the value of this approach is 2-fold. On the one hand, the use of a semi-permeable microdialysis membrane with an appropriate molecular weight cut-off avoids the presence of macromolecules or lipids in the dialysate. On the other hand, NMR profiling can be performed using a sensitive NMR detection coil optimized for the collected dialysate. In recent studies (Glöggler et al., 2016; Crémillieux et al., 2018), we reported the feasibility of using NMR microsolenoids, with inner volumes ranging from 1 to 2 μL, for an online quantitative assay of extracellular brain metabolites in healthy and glioma-bearing rats. Thanks, among other things, to the optimization of the microcoil-filling factor and to the reduction of magnetic susceptibility effects, the limit of quantification (signal-to-noise ratio>10) for lactate was lowered to 1.2 nmol/$\sqrt{}\min$, corresponding to a lactate concentration of 0.6 mmol.L^−1^/$\sqrt{}\min$ in the dialysate. These values were compatible with the concentration of lactate collected in the microdialysate and allowed us to measure online the variations of lactate concentration during the administration of a LDH inhibitor.

As an extension of this previously validated MRS protocol, we investigated in the present study the use of a microdialysis membrane positioned in the rat barrel cortex, combined with a sensitive NMR detection microcoil wrapped around the microdialysis tube at the exit of the microdialysis probe to assess, online, during neuronal activation, the variations of lactate concentration in brain ECF.

## Materials and Methods

### Animal Preparation

Six-weeks old male Wistar rats (*n* = 8; 270 ± 26 g) were purchased from Janvier Laboratory (Le Genest-Saint-Isle, France) and kept in standard housing conditions (12 h light-dark cycles) with a standard rodent chow and water available *ad libitum*. Before the beginning of the experiment, the animals were acclimatized in a temperature-controlled environment for 1 week. Experiments were performed according to the national regulations and were approved by the research ethics committee of the University of Bordeaux (reference number 04490.02).

Sterile surgical procedures were used during the implantation of the microdialysis cannulae (CMA microdialysis AB, Solna, Sweden). Rats were deeply anesthetized with 2.5% isofluorane in a mixture of air/O_2_ (70:30) via a facial mask and positioned in a stereotaxic frame. The head was shaved and the skull's skin was incised in the coronal plane between the ears, along the interaural line. Four small holes were drilled in the skull; two using a 0.8 mm diameter drill bit (for cannulae, −2.5 mm anterior/posterior to bregma, 5 mm lateral to bregma) and two others with a 1.3 mm drill bit (for screws, 3 mm near the previous holes). Two cannula guides were placed 1 mm deep in the smaller holes, located above the left and the right barrel cortex (S1BF area). The cannula placed on the right side was used as a backup cannula in case of a problem on the left side (bleeding when inserting the microdialysis membrane for instance). Plastic screws were inserted in the largest holes. Screws and guides were fixed on the skull with dental glue and then muscles and skin were sutured. Following the surgery, animals were housed individually and received doses of buprenorphine 0.05 mg/kg every 12 h for 48 h.

### Solenoidal Microcoil

The RF transmitter/receiver ^1^H solenoidal microcoil was built in house at the University of Bordeaux. The 220 μm thickness copper wire was hand-wrapped (17 windings) around a polyamide tube of inner and outer diameter equal to 750 and 780 μm, respectively. The microsolenoid was 4.8 mm length and its inner detection volume was 2.1 μL. The filling factor of the microcoil was 93%. The microsolenoid and the polyamide tube were placed vertically in a 2.5 cm length polyethylene cylinder filled with 1.15 mL of Fluorinert FC-70 (Sigma-Aldrich, L'Isle d'Abeau, France) to eliminate air-probe interface. Coil tuning and matching were performed with variable capacities mounted on an adjacent circuit board. The connecting wires between the solenoid and the tuning capacity were 12 mm long. The quality factor (the resonance frequency over the full width at half maximum of the reflection coefficient) of the loaded coil positioned within the MRI magnet was 52 and the full width at half maximum of the water peak was 7 Hz.

### MRI/MRS and Stimulation Protocol

**Figure 1:**
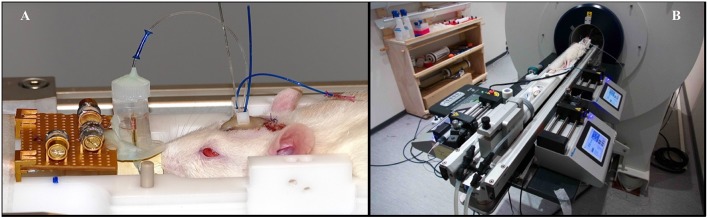
Illustration of the experimental set-up. **(A)** The slightly anesthetized animal is lying prone with a perfusing solution flowing through the cerebral-implanted microdialysis probe. The dialysate is flowing through the microsolenoid NMR coil for online MRS measurements. The overall experimental set-up is shown on **(B)**, with two syringe pumps for medetomidine administering and microdialysis perfusion. To avoid hypothermia, animal is lying on a heated MRI bed.

The MR experiments took place 48 h after the cannula was installed, allowing the animal to recover from surgery. Animals were anesthetized with i.v. perfusion of medetomidine (100 μg/kg/h) and positioned prone on the MRI bed. The microdialysis probes (CMA 7 metal free, microdialysis AB, 1 mm long, 6 kD cutoff) were placed inside the guide cannulae. The inlet and outlet of the microdialysis were connected to a syringe pump (Model 100, KD Scientific Inc., Holliston, USA) on one side (inlet) and to a 130 mm tube (Phymep, Paris, France) of 120 μm inner diameter on the outlet part. The extremity of this tubing was fastened to the upper part of the polyamide tube supporting the microsolenoid NMR coil. An overview picture of this experimental set-up is presented in Figure 1. Microdialysis solution was perfused at a rate of 0.25 μL/min and consist in an isotonic artificial cerebrospinal fluid (aCSF) solution (NaCl, 124 mmol/L; KCl, 3 mmol/L; CaCl_2_, 2 mmol/L; MgSO_4_, 1 mmol/L; KH_2_PO_4_, 1.25 mmol/L; NaHCO_3_, 26 mmol/L; pH 7.4) containing also 1.5 mmol/L [Gd^3+^] (gadoterate meglumine, Dotarem®).

MRI and MRS acquisitions were performed at 7 T (Bruker, Biospec 70/20). A transmitter linear coil and a rat head surface coil array 2 × 2 for reception (Bruker) were used to control microdialysis probe location in the S1BF area and the proper functioning of the microdialysis membrane. Anatomical T_1_-weighted MR images were obtained with a RARE (rapid imaging with refocused echoes) spin echo sequence (matrix size, 256 × 256; number of slices, 12; slice thickness, 1 mm; repetition time (TR)/echo time (TE) = 600/10 ms; total acquisition time, 4 min 10 s).

For proton MRS acquisitions, the microsolenoid coil was placed at the isocenter of the magnet and connected to the preamplifier of the MR spectrometer. Shimming was performed using an automatic first and second-order shimming procedure. MR proton spectra obtained with the microcoil were acquired using a single-pulse sequence (TR, 2.5 s; acquisition bandwidth, 3 kHz; spectral resolution, 1 Hz/point; excitation pulse length, 7.5 μs; excitation pulse power, 0.4 W; 100 averages) corresponding to an acquisition time of 4.2 min per spectrum.

Spectra were collected continuously, alternatively at rest and during stimulation (40 and 15 min, respectively). Activation of the left barrel cortex was obtained by the stimulation of right vibrissae of the animals with an amagnetic air-pulsed system using a paradigm to avoid neuronal desensitization (8 Hz, 20 s activated/20 s rest) as previously published (Mazuel et al., 2017).

As a point of comparison, an *in vivo* localized proton spectrum was acquired at rest on an animal without implanted microdialysis in a voxel located in the S1BF area (2 × 2.5 × 3 mm) using a PRESS sequence (TE, 20 ms; TR, 2,500 ms; 256 scans; total acquisition time of 10.7 min) as described in a previous study (Mazuel et al., 2017).

For some animals (*n* = 2), dialysates collected separately in Eppendorf tubes during rest and activation periods were further analyzed on a 500 MHz Bruker spectrometer (Ascend, Bruker, Wissenbourg, France). Dialysates were diluted in a 440 μL solution of deuterium oxide containing 0.75% (w/v) trimethylsilylpropanoic acid (TMSP) as a reference (Sigma-Aldrich Chemie, Saint-Quentin Fallavier, France). Proton spectra were obtained using excitation sculpting to suppress water (Zgespg sequence from the Bruker pulse library; acquisition bandwidth, 5 kHz; relaxation delay, 1 s; number of averages, 128). Spectral analysis (peak identification and quantification) was performed using Bruker software TopSpin 3.1. The amplitudes of the meglumine peaks in the perfusate and dialysate (obtained after 30 min of perfusion) were calibrated with TMSP used as an internal reference of known concentration.

At the end of the experiment, rats were euthanized using an overdose of anesthetics.

### Statistical Analysis

Comparison of lactate peak integrals between rest and stimulation periods was performed using paired *t*-test with two-tailed *P*-values. Significance was fixed at a 1% probability level. All analyses were performed using GraphPad Prism (GraphPad Software Inc., La Jolla, CA, USA). All the data are presented as the arithmetic mean value ± standard deviation (*SD*).

## Results

**Figure 2:**
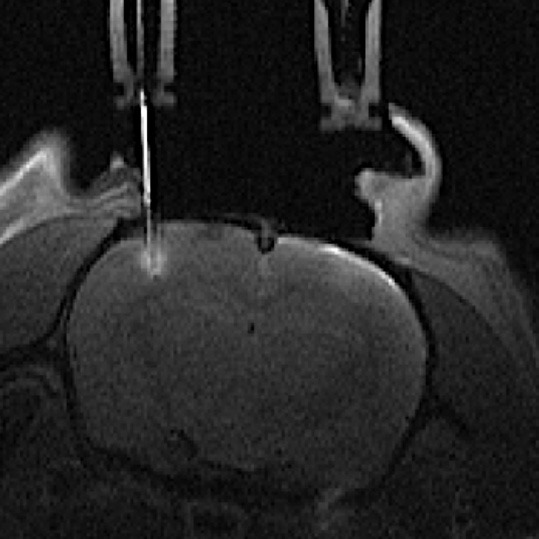
Axial T1-weighted MR image of a rat brain with one microdialysis probe located on the left side barrel cortex. The gadolinium chelate added to the perfusion solution is highlighting the MRI signal inside the microdialysis probe and within the tissue surrounding the semi-permeable membrane.

From T_1_-weighted RARE images, microdialysis membrane locations in the barrel cortex were easily and accurately assessed (Figure 2) due to the gadolinium present in the dialysate perfusion solution, which leads to tissue enhancement in the vicinity of the semi-permeable membrane.

Meglumine concentrations, measured using the 500 MHz NMR spectrometer and calibrated with TMSP, were found to be identical in the perfusate and in the dialysate (after 30 min of perfusion) and equal to 1.5 mmol/L. These results reflect the equilibrium conditions of meglumine exchange through the microdialysis membrane obtained after 30 min of perfusion.

**Figure 3:**
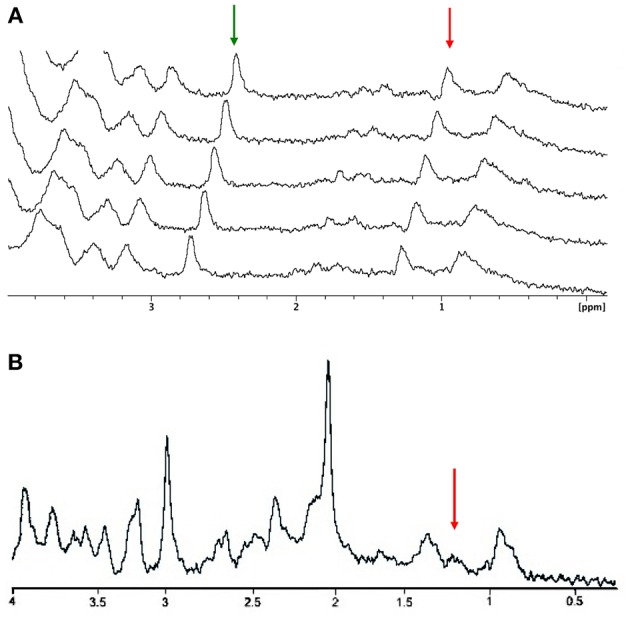
Resting MRS spectra. **(A)** Stacked plots of consecutive microcoil magnetic resonance spectra obtained under steady-state conditions. The amplitude of the meglumine peak (2.8 ppm; green arrow) and lactate methyl peak (1.3 ppm; red arrow) are stable along the rest period. **(B)** Localized proton spectrum obtained in the barrel cortex. The red arrow is indicating the lactate methyl peak.

A consecutive series of microsolenoid NMR spectra on the microdialysate from the probe located in the barrel cortex region are shown in Figure 3A. Spectra were acquired before whisker stimulation got started. Two NMR peaks are identifiable on these spectra; the peak of the methyl proton of meglumine (counterion of the gadoterate) at 2.8 ppm and the peak of lactate methyl protons at 1.3 ppm. Integrals of these two NMR peaks are stable during successive acquisitions, illustrating steady state conditions. A ratio of 0.47 ± 0.06 was found between integrals of lactate and meglumine peaks. Since the meglumine concentration is known (1.5 mmol/L), the lactate concentration was estimated to be 0.7 ± 0.1 mmol/L in the dialysate from the barrel cortex, during the rest period in this particular example. The average lactate concentration measured before stimulation for all animals was 0.6 ± 0.1 mmol/L.

An example of *in vivo* localized ^1^H NMR spectrum obtained in a voxel localized in the entire cortex barrel is presented in Figure 3B. Despite the longer acquisition time (10 vs. 4 min with the microcoil), detection and quantification of the lactate peak appear more challenging due to low signal intensity and overlap with lipid signals (the lactate peak appears as a shoulder on the right part of the lipid peak).

**Figure 4:**
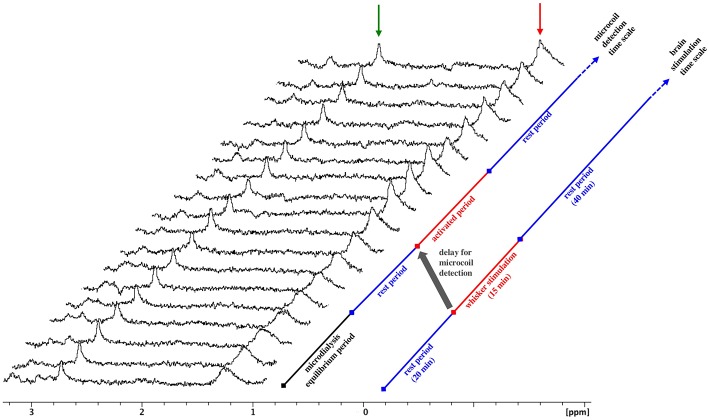
MRS spectra upon whisker activation. Stacked plots of consecutive microcoil spectra, time increasing from bottom to top, obtained during whisker stimulation. Increasing amplitude of the lactate peak is observed from spectrum #9 to spectrum#12, corresponding to the dialysate collected during the stimulation period. Red arrow: lactate, green arrow: meglumine. The gray arrow highlights the time lag between the stimulation period and the detection of amplitude change.

A series of consecutive spectra obtained during a rest-stimulation-rest protocol is presented in Figure 4. The amplitude of the meglumine peak is constant in all spectra, reflecting the equilibrium conditions for gadoterate meglumine exchange through the microdialysis membrane throughout the experiment. In contrast, following whisker stimulation, an increase in lactate methyl peak intensity is observed, followed by a slow decrease. Approximately 20 min elapse between the start of whisker stimulation and the detection of the lactate peak increase. This time lag corresponds to the time required for the dialysate to travel the distance from the microdialysis membrane to the NMR microsolenoid. Measurement of this transit time was reported in a previous study (Crémillieux et al., 2018). This increase in lactate was observed in five consecutive spectra, a time window that corresponds ~15 min whisker stimulation period.

**Figure 5:**
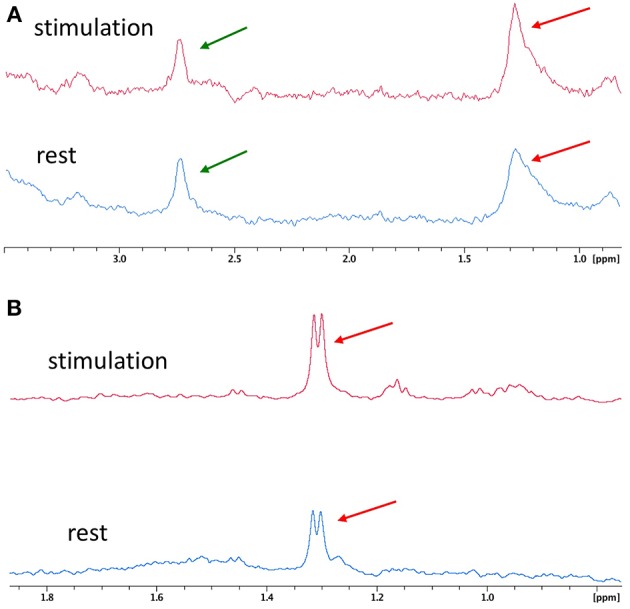
Typical ^1^H MR spectra between rest and whisker activation. **(A)** ^1^H spectra of microdialysates from rat #7 obtained online with microcoil during rest (blue, bottom spectrum) and stimulation (red, top spectrum) periods. **(B)** Corresponding high resolution spectra from rat #7 obtained on a 500 MHz Bruker magnet from the collected dialysates and centered on lactate proton methyl peak during rest (blue, bottom spectrum) and stimulation (red, top spectrum) periods on rat#7. Red arrow: lactate, green arrow: meglumine.

Figure 5 compares two spectra obtained during the rest period and the stimulation period with the microsolenoid (Figure 5A) and the corresponding high-resolution 500-MHz spectra (Figure 5B) obtained on the dialysates corresponding to a rest and a stimulation period. Based on their amplitudes relative to the meglumine peak, the upper limit for lactate concentration in the dialysate was evaluated to be equal to 0.23 and 0.37 mmol/L during rest and activation period, respectively, corresponding for this specific acquisition to a 47% increase in lactate concentration during neuronal activation in the barrel cortex. A similar, although weaker, 27% increase in the lactate peak integral (methyl proton doublet) between rest and stimulation period is observed when using the high-resolution spectrometer on the dialysate.

**Figure 6:**
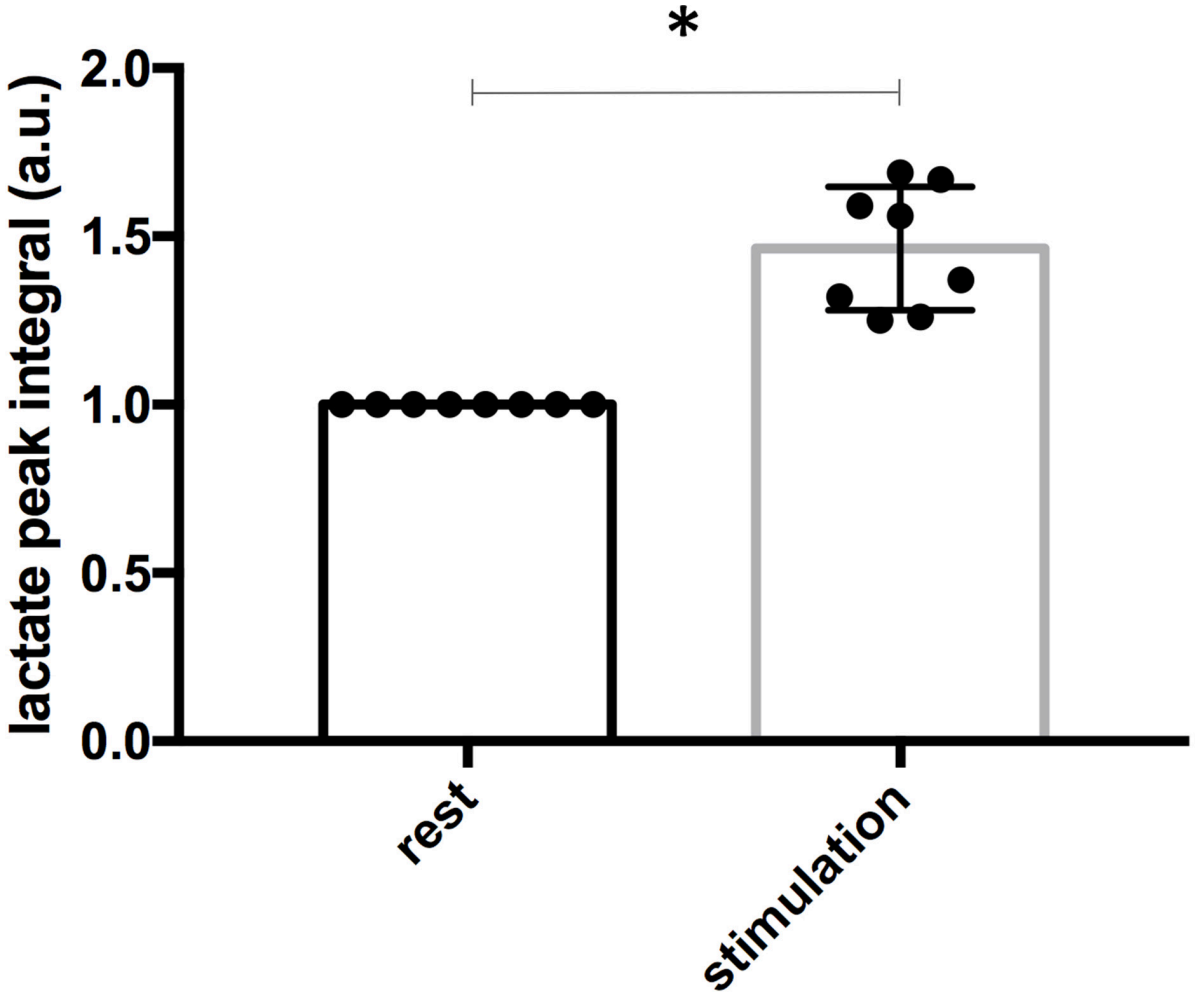
Quantification of lactate signal in resting and activated conditions. Lactate concentrations were quantified by lactate methyl peak integrations using meglumine peak as internal reference. Values in the stimulation period were normalized to the one measured in the rest period for each animal (*n* = 8). Asterisk indicate significant differences (*P* < 0.01). Data are presented as the arithmetic mean value ± standard deviation (*SD*).

As shown in Figure 6, the average increase in the amplitude of the lactate peak measured with the microcoil between the rest period and the stimulation period was 37 ± 11% (*n* = 8). This change in amplitude represents a statistically significant increase (*p* = 0.0002) in the extracellular lactate concentration during whisker stimulation.

## Discussion

In a recent study (Crémillieux et al., 2018), we showed that, in the absence of macromolecules and lipids in the microdialysate, an accurate determination of lactate levels both in a glioma and in the healthy brain could be obtained online using a sensitive NMR microcoil. In this previous study the detection and quantification of lactate was, however, performed under isoflurane anesthesia, which is known to highly increase brain lactate concentration (Horn and Klein, 2010).

As an alternative to anesthesia, MRI or MRS experiments can be performed on awake rats but this protocol requires the animal to be accustomed to a stressful immobilization in the confined and noisy environment of the MRI scanner tunnel. Considering the use of anesthetics as a necessary evil for this MRI and MRS study and bearing in mind the interference of anesthetics on brain physiology and function (Masamoto and Kanno, 2012), the *in vivo* NMR protocol was performed using α2-adrenergic agonist medetomidine. Indeed, volatile anesthetics interfere with mitochondrial energy metabolism by inhibiting complex I (Muravchick and Levy, 2006), which leads to uncoupling between glycolysis and oxidative metabolism and thus lactate formation. Medetomidine, together with dexmedetomine and xylazine, are α2-adrenergic agonists, which have been shown to have the lowest impact on brain lactate levels (Horn and Klein, 2010), brain blood flow (Lawrence et al., 1996; Weber et al., 2006), and plasma glucose concentration (Sinclair, 2003).

Using this protocol, lactate content was at its physiological concentration, in the mmol/L range. Even under this low concentration condition, its NMR peak was easily detectable using our microcoil with a signal-to-noise ratio (SNR) compatible with the desired limit of detection (SNR > 3) and limit of quantification (SNR > 10).

We took advantage of the presence in the perfusate of meglumine, an NMR detectable molecule, for monitoring the stability of the experimental conditions. Its detection was used to reach steady-state condition before starting the experiment. Since its concentration was known, meglumine peak was also used for lactate absolute quantification.

In the absence of whisker stimulation, the average lactate concentration in the dialysate was 0.6 mmol/L. The recovery rate, ratio of the concentration of a given metabolite in the dialysate to its extracellular concentration in the tissue, depends on multiple parameters, such as the molecule itself, the physical length of the microdialysis probe, the cutoff of its semi-porous membrane and the rate of the perfusate flow in the microdialysis. *In vivo* recovery rate for lactate was previously reported in the literature and values were 10 and 30% for perfusate flows of 1 and 0.5 μL/min, respectively (Boubriak et al., 2006; Caesar et al., 2008). Assuming a 50% recovery rate for a perfusate flow of 0.25 μL/min, the extracellular *in vivo* lactate concentration would then amount to 1.2 mmol/L, a value in good agreement with the one measured *in vivo* by microdialysis (Abi-Saab et al., 2002).

As mentioned in the introduction, accurate NMR measurements of lactate concentration are quite difficult to obtain due to the small amplitude of the lactate peak and its overlap with other large proton resonances. Nevertheless, several groups investigated lactate content changes *in vivo* during brain activation using ^1^H-MRS, but this was measured mainly in human (Prichard et al., 1991; Sappey-Marinier et al., 1992; Mangia et al., 2007; Bednarík et al., 2018). Indeed, it becomes much harder to perform this kind of study in the rodent brain, where voxel size is much smaller. However, it would be of great interest to be able to follow lactate changes in pathological rodent models and transgenic animals. To overcome these difficulties, Mazuel et al. (2017) used focused microwave to fix rat brain just after whisker stimulation and performed *ex vivo* ^1^H- and ^13^C-NMR analyses of the barrel cortex. Using this technique, a 44% increase in lactate concentration was reported in this somatosensory area following whisker stimulation. Direct *in vivo* ^1^H MRS was also performed by Mazuel et al. but the lactate peak was difficult to quantify. The observed variations of lactate concentration are in line as well with previously reported variations of lactate using microdialysis sampling and enzymatic assay of lactate (Caesar et al., 2008). In this study, Caesar et al. showed that that climbing fiber stimulation of cerebellar Purkinje cells in rats increased extracellular lactate by 30% within 30 s of stimulation.

Lactate quantification in a small volume (few microliters) of dialysate fluid are usually performed remotely from the animal using high-sensitivity analytical techniques, such as biochemical assays, chromatography, mass spectrometry or high resolution nuclear magnetic resonance (Kennedy, 2013). The use of these analytical techniques is compatible with experiments in unanesthetized and freely moving animals but in practice the application of a protocol such as whisker stimulation requires the use of anesthetics, in particular with imaging techniques such as *in vivo* MRI where acquisition on awake animals requires lengthy habituation of the animal to prolonged immobilization in the MRI scanner tunnel. Owing to the complexity of most of these techniques, their temporal resolution is generally superior to several minutes and prevents the investigation of real-time changes in lactate concentration (Kennedy, 2013). This being said, Caesar et al. took advantage of the high detection sensitivity of enzymatic assay and implemented on-line measurements of lactate concentration in brain microdialysate with a remarkable 12 s temporal resolution in anesthetized rats (Caesar et al., 2008).

Although the microcoil approach does not allow at the moment such a high measurement rate, the time resolution of the protocol (currently few minutes) can easily be reduced by several protocol optimizations; (i) dead volumes and transit times of the microdialysate can be reduced to almost zero by positioning the microsolenoid directly at the output of the microdialysis probe, (ii) detection sensitivity can also be improved by further reducing the flow of the perfusate to increase lactate exchanges and reach nearly equal lactate concentrations between extracellular space and microdialysate, and (iii) performing this protocol at higher magnetic Bo field would improve lactate detection, since SNR of resonance lines increase with Bo^7/4^.

The microcoil detection protocol offers several peculiar and unique characteristics. It gives the possibility of acquiring highly localized NMR spectra and MRI images with the same NMR equipment during a single acquisition session. MRI can be used to ensure accurately that microdialysis probe is correctly located (Elmeliegy et al., 2011) and by adding a contrast agent to the perfusate, it also helps to ensure that the membrane works properly. The spectroscopic measurement can be combined as well with BOLD fMRI studies to record the haemodynamic response associated with neuronal activation. By using a molecule (meglumine in this study) detectable by NMR whose concentration in the dialysate is known, it is possible to achieve an absolute quantification of the lactate concentration.

Although this study focused on the proton NMR detection from lactate, the technique can be adapted for ^13^C spectroscopy by using microcoil tuned to ^13^C Larmor frequency, as reported in a previous study (Glöggler et al., 2016). The protocol could then be used with local administration of ^13^C-labeled substrates ([1,6-^13^C_2_]glucose for instance) in the perfusate followed by online detection by ^13^C MRS of metabolites such as ^13^C-lactate formed from this labeled precursor. One can also think of the use and detection of hyperpolarized ^13^C-labeled substrates (Duckett and Mewis, 2012; Hurd et al., 2012) administered to the animal either systemically or in solution within the perfusate.

In summary, this study presents the first online MRS measurements of changes in lactate concentration induced by brain stimulation in an animal model. These initial results represent a first step toward more elaborate protocols combining localized MR spectroscopy and MRI for a better understanding of neuroenergetics and brain metabolism.

## Data Availability

The datasets generated for this study are available on request to the corresponding author.

## Author Contributions

YC conceptualized the study. YC, UD, LM, RS, VZ, SR, and NP participated to the MRI and MRS investigations with the microcoil and to the corresponding data analysis. NP realized the NMR microcoils used in the study and performed the high-resolution NMR analysis. JB, LM, HR, and A-KB-S performed the MRS investigation with the surface coil and the corresponding data analysis. HR, JB, LM, and A-KB-S provided the support and guidance for the experimental animal protocol and the implementation of the whisker stimulation protocol. YC and A-KB-S wrote the manuscript with comments from all authors.

### Conflict of Interest Statement

The authors declare that the research was conducted in the absence of any commercial or financial relationships that could be construed as a potential conflict of interest.
